# Bourdieu and programming classes for the disadvantaged: a review of current practice as reported online—implications for non-formal coding classes in Bali

**DOI:** 10.1186/s41039-018-0068-x

**Published:** 2018-03-27

**Authors:** Laurence Tamatea, Gusti Agung Ayu Mas Pramitasari

**Affiliations:** School of Education, Charles Darwin University, Casuarina, Australia

**Keywords:** Software development, Poverty, Disadvantage, Programming, Bourdieu, Bali, Baudrillard, Coding, Indonesia

## Abstract

The software development sector is rapidly expanding. It generates vast revenues whether categorised under ICT or creative industries. It has a capacity to support national economic growth and is supportive of economic development. Despite such opportunity, the poor and disadvantaged are often excluded from access to and participation in this sector. In light of this context, our paper comprises a first step in a project aiming to offer software development classes to disadvantaged youth in Bali, Indonesia. To guide construction of the curriculum, we review publically available online literature around current practice in the provision of non-formal education-based programming classes to the disadvantaged. We wanted to know what might constrain disadvantaged students from participation in this field, what as a consequence might be their response to educational opportunities for this field, and what might be needed to provide ‘agentic’ learning experiences. We also wanted to evaluate the suitability of Bourdieu’s Social Reproduction Theory in terms of reading this literature and providing a broad level framing to guide initial thinking about curriculum construction in response to these questions. This paper presents findings from our review of the online publically available literature and argues the value of Social Reproduction Theory to understanding current practice and guiding preliminary thinking around curriculum construction.

‘IDC estimates there were 21.03 million software developers in the world at the outset of 2017,’ said Arnal Dayaratna, research director, Software Development at IDC. ‘IDC estimates that 11.13 million are full-time developers, 5.77 million are part-time developers, and 4.13 million are nonprofessional developers. The Asia/Pacific region accounts for 9.47 million of the world’s developers, while the EMEA and Americas regions represent 6.76 million and 4.80 million, respectively. Notably, China and India are responsible for 31.5% of the world’s total developer population’ (Dayaratna 2016).

## Introduction

Employment websites reveal high demand for those with information communications technology (ICT) skills. On 7 November 2017, the software Development category at the seek.com (2017a), for example, sat comfortably within the top ten in terms of job vacancies in a list of approximately 50 categories. At https://www.seek.com.au/ where software development is located in the ICT category, it ranked highest, offering over three times the number of jobs as second ranked category, construction. Software developers seemingly have ample employment opportunities. The strength of the field continues in the relationship between growth in the ICT and software development sector and regional and national economic growth; a relationship which mostly holds even for ‘developing’ countries, with India perhaps as the best example (Arora and Gambardella 2008; 2005). But as positive as these indicators seem, we ask how individuals from disadvantaged backgrounds fare in this relationship, particularly those in ‘third-world’ or developing contexts? Here we refer to Bali specifically, which despite association with ‘paradise’ and a tourism industry attracting millions of tourists, remains very much a third-world contexts inscribed by socio-economic disadvantage (Sapsuwan 2014; The Bali Times 2012a; Surbakti 2012). Our experience of Bali is that life in a rural village or urban slum can be far removed from the world of software development, not only in terms of access to hardware and software, but also in terms of access to the field’s socio-cultural properties. A disposition towards employment in software development is not easily developed or maintained when there are more pressing short-term needs (Marinos 2016; Kayser 2013).

Yet some, whose lives are framed by poverty, succeed. There are, for example, ‘graduates’ from the Balinese-owned NGO we work with in Bali, the *Slukat Learning Centre* (SLC), who from poor rural village backgrounds have become what would have once been unimaginable. Students from SLC (Slukat Learning Center 2017) now study in universities around the world. Others have achieved similar outcomes within Indonesia; some enrolling in Masters Programs, while others have established careers beyond servicing the tourist industry. The exercise of agency leading to change and success, it seems, is possible. The Slukat Learning Center does not, however, offer a software development curriculum (also referred to hereafter as coding), a gap to which our project responds. We aim to develop and implement a coding curriculum for disadvantaged rural Balinese youth who attend the Slukat Learning Center. Though in Bali there are indeed those who are already well positioned by and within the (‘Western’) global capitalist economy as manifest at local and national levels (Jaya Guna 2017, Bali Rich List 2013), the distribution of wealth remains uneven, such that for the ‘poor’ and disadvantaged, a key goal and indicator of success is simply to increase their income (Mitchel 2014). Consequently, our project does not so much aim to equip students with the capacity to alter, challenge or even subvert the global economy or its national/local expression (that is another project altogether). Rather, it aims to provide disadvantaged students with skills valued by this economy, which may enable them to generate income in a (software development) field otherwise considered simply out of reach. This of course, is a goal which cannot be easily dissassociated from the broader global and national context within which it is set, in so far as this relates to growth in the significance of softwre development. This is a context we argue, which offers opporunities for enhanced agency and success to those who hold the right skills set.

Although, within this context, Indonesia and indeed Bali are neither internationally significant nor top-tier software development centres, it is, we argue, almost inevitable that this sector in Indonesia (and in Bali) will continue to grow, such that those equipped with coding skills *may* be well positioned to take advantage of this. We understand this 'ínevitibility' from a theoretical perspective, and from the intensity of the current trajectory in the growth of the software development sector. Regarding the theoretical we look to the work of Baudrillard (1995) and see ínvitibility arising from a key dynamic of our times, which is abstraction. This dynamic, which arguably underpins the economic and statistical evidence noted below, facilitates understanding why now the economic case for a programing curriculum for the disadvantaged should be so significant. Though much of Baudrillard’s work is concerned to account for our televisual world in terms of its functioning, dynamics, and impact upon knowledge, society and liberty, it is his claim that humanity has entered a fundamentally different paradigm that is of most relevance to this discussion. In this paradigm interaction with the world, self and others is increasingly mediated by digital televisual technologies including cybernetics, computers and the Internet (Baudrillard 2005, 2002, 1995, 1993). In elaborating this claim, Baudrillard provides an historic trajectory, mapping humanity’s arrival at its current position with regard to representation and engagement with the world. Our view is that software development is strongly representative of the ‘nature’ of the third order of simulacra (within this trajectory), which is one of intensifying abstraction (Baudrillard 2005).

Baudrillard maintains (1995) that there have been three orders of simulacra following the original condition of symbolic exchange, each grounded in a relationship between humanity, the image (as in re-presentation) and reality. The initial condition of symbolic exchange comprised a time when reality was taken to be fixed and God-ordained. Imagery functioned to reproduce the divine order of the real, with the image being ‘a good appearance’ of the real, thus of a ‘sacramental order’ (p. 6). The question that reality might be otherwise, that it could be re-presented otherwise was a ‘non-issue’. Imagery functioned to dissimulate or ‘to pretend not to have what one has’ (p. 3). In the second order of simulacra, a shift takes place, from dissimulation to simulation. In contrast to dissimulation, the move to simulate is to ‘feign to have what one doesn’t have’ (p. 3). In the second order, roughly from the Renaissance to the mid-twentieth century, industrial technologies impact image production, including (analogue) photography leading to the mass production and re-production of copies. By contrast, the third order is ‘founded on information, the model, the cybernetic game—total operationally, hyper-reality, aim of total control’ (p. 118). This is our time, our order; the one from which digitisation and software development arise.

In this third order, the image may have ‘no relation to any reality whatsoever’ (Baudriallard, 1995, p. 6) and, as realised through digital technologies, can be pure simulation (2005). What is more, simulation is reabsorbed feeding back into the real, creating a hyper-reality (1995, p. 118) wherein reality engagement is through a (digital) model against which reality itself is evaluated. Simulations reference other simulations, while the real lies ‘rotting’ in the corner (p. 145). With the intensification of digitisation, the territory no longer precedes the map, nor does it survive it—‘welcome to the desert of the real’ (p. 1) as Morpheus (quoting Baudrillard) explains to Neo in the Matrix (Warner Bros 1999). Today, Baudrillard asserts, abstraction is increasingly the means through which we access real. Software developers, it seems, are at the epicentre of this intensifying dynamic. While this may seem overly fanciful if not overly theoretical, Edell whose business involves ‘helping companies succeed with machine learning’ puts this very simply: “Everything today is abstracted. Our grocery stores abstract growing food. Our roads and transportation methods abstract travel. Our devices abstract communications” (2016). Baudrillard’s model not only provides a frame for understanding why there are so many software developer job vacancies, it arguably provides a frame for understanding the figures pertaining to revenue generated by the ICT field more broadly (including spending on hardware, software and services), and within this software development. Here we reference software development both as a ‘field’ in the general sense of an area of human endeavour, and also in terms Bourdieu’s Social Reproduction Theory, in which the notion of field is central. While Bourdieu’s conceptualisation of the field is treated in more detail below, the value which Bourdieu adds to understanding the software development field as a site to which the disadvantaged might gain access, is we argue, derived from Bourdieu’s recognition that a field is always inhabited by individuals in a relationship with others who are framed by its rules of the game or doxa (Bourdieu and Wacquant 1992, p. 101).

Though reports vary regarding revenues in this field, they are, however, constantly impressive. Statistica, for example, estimated that in 2017 global spending on enterprise software alone was expected to reach 351 billion U.S. dollars, ‘while IT services, the second largest segment behind communications services, [was] set to reach 922 billion U.S. dollars’ (2017); others point to even higher numbers (ETCIO 2016). Elsewhere, Chatterjee (2014) claimed the size of the global IT services market to be US $850 billion, globally, while as early as 2003, Askari and Chaterjee (2003) were predicting ‘worldwide markets for professional IT services [would] break the $1 trillion mark within the next two years, growing from an estimated $462 billion in 1999 to about $1.08 trillion by 2004, at an 18.6 per cent Compounded annual growth rate’^1^. With increasing consumer demand and business competition (Bartels and Giron 2017; Carlson 2006), ‘companies that heretofore had little to do with technology are digitising their products and services with software to deliver more value to customers’ (PwC Technology Institute 2014; Arora et al. 2013). Software development earnings can also be situated among those of the creative economy, which as early as 2000 was generating ‘US$2.2 trillion worldwide’ and growing at ‘an annual rate of 5 per cent’ (Boccella and Salerno 2016).

Others have also acknowledged what seem promising business opportunities in Indonesia arising from the use of digital technologies (Hasan et al. 2016). An International Data Corporation (IDC 2016) Indonesia ICT Market Landscape study reported that as of 2016 with Indonesia’s Gross Domestic Product (GDP) growth of 4.73% (p. 1), and Foreign Direct Investment at a rate of 18%, ‘global companies are seeing high market potential in the country’ (p. 1). While largely confirming statistics reported elsewhere, indicating ecommerce in Indonesia was expected to ‘hit $10 billion by 2016, being the economy’s “fastest growing sector”’ (MKK 2015), of significance in these is that ‘consumer spending makes up slightly more than 50% of IT spending in the country, driven by consumers who are continuously purchasing mobile devices, PCs, and printers’ (IDC 2016, p. 1). These statistics point to the emergence of a strengthening consumer product development integration which supports software development sector growth (Arora 2006). While IT service providers in Indonesia are dominated by ‘global players’, local firms are leveraging a capacity to work with local cultural orientations (p. 2) such that as Sumirat et al. note with reference to the Indonesian start up Go Jek, the digitisation of Indonesian society has ‘strengthened the platform for [a] blossoming online creative economy’ (Sumirat et al. 2016).

For Indonesia the relevance of ICT and indeed software development to national economic development is recognised in the establishment of the Indonesian organisation for the creative economy—Badan Ekonomi Kreatif Indonesia (BEKRAF).^2^ President Joko Widodo has mandated that Indonesia will be a world leading power in the creative economy by 2030 (Sumirat et al. 2016), while Hari Santosa Sungkari the BEFRAF deputy for Infrastructure expects that by 2019 there will have been a ‘12 per cent surge in the creative economy’s contribution to the country’s gross domestic product’ (Singgih 2017). While BEKRAF comprises 16 sub-sectors, linked to ICT broadly and software development and services more specifically, it is interesting to note that one of these is software applications and game development (aplikasi dan pengamban mainan). BEKRAF maintains that with:

“Increasing penetration, public use cannot be disconnected from the role of applications in this. People are already well versed in using various kinds of digital applications like maps for navigation, social media, news, business, music, translators, games and so on. Some of these applications are designed to make daily life easier. Consequently, it is not surprising that there is a big potential in this sub-sector” (2017).^3^

While we return to this statement later, it is valuable in this acknowledgement of the context to also acknowledge the coding environment in Bali, which will be the more immediate environment framing the proposed coding curriculum. Though like Indonesia more generally, Bali is not considered to be in the top three tiers of software development, it remains nonetheless inscribed by a solid range of digital flows and is gaining a reputation as a tech start-up center (Loubier 2014). As Noviandari states:

“Bali is a well-known paradise for tourists, treating them to its beautiful beaches, volcanoes, scenic rice fields, great food, and an abundance of adventurous island activities. What you may not know is that Bali has a fast growing startup scene. There are many entrepreneurs, initiatives, events, startups, and some tech talent based on the island” (2015).

While Denpasar^4^ is now an official ‘smart city’ (AntaraBali 2017), Bali itself is home to a number of code ‘bootcamps’ such as the Institute of Code, Le Wagon, The Sanur Space and Coding Nomads. Hubud (located in Ubud), for example, not only teaches coding but provides opportunities for professionals to network, co-work and conduct (digital) business. There are also a number of informal and formal coding groups such the Google Developer Group Bali. Indeed, that Livit (which provides startup studio spaces) has a branch in Bali (in addition to Zurich and Denmark) is indicative of Bali’s growing status as a coding setting (and market). Bali also has numerous local web development/design businesses such as Slinky Web Design, DigiBali, Pantun Studio, Bali Website Videos, BaliGatra and BaliWebPro to name a few. And in terms of connections with larger corporate software development, Mitrais exemplifies the capacity for Bali to be a hub within a much larger regional and international software development enterprise (Earl 2016). Jakarta, of course, is home to more corporate entities and regional and global links, which is largely a product of its market size, being the base of operation for a diverse range of industries requiring software. Nonetheless, a cursory review of job vacancies in Bali let alone Indonesia more broadly, also shows a healthy demand for developers. At the time of writing, Livit required the following in Bali: Technical Artist, C# Content Developer, Senior C# Software Engineer, DevOps Engineer, and a Python Developer (Livit 2017).

Based upon the apparent strength of this context and the possibilities it seems to offer those with the approprate skills internationally and in Indonesia, our paper comprises a first step towards development of a coding curriculum for disadvantaged youth in Bali, to be implemented at the SLC. In this paper, we review the publically available online literature reporting current practice in providing coding classes to the disadvantaged. The literature is read through the lens of Bourdieu’s Social Reproduction Theory. Though in developing our coding curriculum we hold to the liberal-humanist (individualist) tenant that all are equal and free to choose their own version of the good life (Tamatea 2016), we understand this ideal to be qualified by the context in which individuals and groups are located. Hence, as noted, we draw upon Bourdieu to identify the extent to which current practice, as reported in the publically available online literature, can be accounted for using his Social Reproduction Theory, as a precursor to using this theoretical framework to guide initial reflection upon constructing our coding curriculum. We argue with reference to the online literature around current practice, that Social Reproduction Theory adequately accounts for existing practice and as such it will be valuable to our project. But our review highlights that a coding curriculum providing disadvantaged students with a capacity to positively change their life, which supports agency, will need to equip students with a range of capitals (economic, social and cultural) as learning code alone is perhaps insufficient to achieve success. Though we acknoweldge the limitations of Bourdieu's thoery and the not insignificant challenges facing software development and ICT-related education in Indonesia more generally, we maintain nevertheless, that if Baudrillard’s thesis holds, then the need for software developers should only increase in Indonesia such that students in our coding class *may* be well positioned to take advantage of this.

## Methods

This paper aims to provide an informed basis for initial reflection upon constructing a coding curriculum for socio-economically disadvantaged youth at a non-formal education setting in rural Bali, Indonesia. A review of formal academic literature reveals a dearth of research around this specific area, a conclusion largely confirmed by the Erasmus+ study, *Non-formal Education Opportunities In the Field of Computer Programming* (2015). Though disadvantage was not a specific focus of this EU-focused study, it concluded with regard to non-formal education’s provision of coding skills to youth that: ‘For the time being, there is lack of knowledge about how many people are involved in non-curricular programming initiatives’ (p. 5). While much has been written around education for the socio-economically disadvantaged (Zhang 2014; Connell 1994), non-formal education generally (Purwanti and Widiastuti 2014), the use of ICT to support non-formal education more specifically (Malgorzata 2017), ICT and development (Heeks et al. 2017; 2014), using ICT to teach the disadvantaged (Feldman et al. 2003; Deloatch 1977), and the challenges of teaching (and learning) programming (Fotaris et al. 2016), there has been little formal investigation of providing coding classes to the socio-economically disadvantaged in non-formal education settings.

Nonetheless, Ruseva and Rissola’s investigation of *the role of non-formal education in teaching coding* (2016), which springboards from the broader movement by governments to introduce coding to all students in formal education, does raise questions about the extent to which this ‘new literacy’ will be accessible to the disadvantaged. Though not a focused investigation of the relationship between disadvantage and coding in non-formal education, Ruseva and Risola recommend that making coding more accessible to the disadvantaged may require both incentivisation and the supply of more teachers. They also argue that although ‘welfare’ workers and volunteers are important to this goal, ICT professionals may value-add in this respect. But although this seems a solid proposition, we would suggest that possession of professional programming skills be combined with sound pedagogical proficiency (Stewart 2015; Curry 2013).

In some respects, the intent of this paper is similar to that of the Erasmus+ report’s project, in that it aims to respond to a gap in the literature. We, like the Erasmus+ project, want to know ‘what is happening’. The Erasmus+ report expresses this intent as ‘diagnosis of non-formal education opportunities in the field of computer programming’ (p. 3). But whereas this report limited its scope to a number of EU member states, the scope of this paper is considerably more global. The report nonetheless identifies the kinds of non-formal education opportunities available and what comprises ‘good’ practice therein, foci both valuable to our project. With regard to the former, the report identified a number of levels of provision of coding education in the non-formal education ecosystem as follows: (1) online coding platforms, (2) coding resources platforms, (3) event-oriented coding and (4) coding communities. And with regard to the ‘winning features of the best practices for learning coding in a non-formal way’, these included (1) hands-on, (2) result-oriented, (3) added social value, (4) role models, (5) fun, (6) community-focused, (7) sensitive to languages, and (8) right balance between top-down and bottom-up management. Additionally, with respect to engaging girls, the report added to this list the following: girl-focused, no jargon and both female and male role models (pp. 23–24). While the Vecchia et al. European Commission sponsored report, *Formal and Non-formal Educational Programmes on Digital Skills and Competences* (2015), recognises a number of these expressions of best practice, it further highlights the importance of establishing links between the learning experience and industry:

The fact that fewer students embark on a career path in the digital sector does not mean that digital jobs are not attractive, it could also simply mean that students are not aware of what exactly these jobs entail. Practice shows that sometimes students have a biased idea about digital jobs as to being difficult and simply not fulfilling (p. 10).

As a response to the gap in the formal literature, our review of the literature thus shifts focus to that found online to identify instances of practice in providing a coding curriculum to socio-economically disadvantaged youth in non-formal education settings. Though accessing online sources has limitations including the inability to more objectively state that matters represented online are in fact true, accessing these sources also has benefits including providing access to a wider range of perspectives which due to the ‘relative’ anonymity afforded online are more likely to be grounded in authentic disclosure (Tamatea 2005a, 2005b, 2008a, 2008b, 2010, 2011a). And in the specific context of Bali, Tamatea’s study of the *Ajeg Bali* movement following the Bali bombings demonstrated the value of accessing the public voice online to understanding contemporary socio-economic practice and discourse (2011b).

Drawing upon online research methodology deployed by Tamatea, the literature introduced below comprise polemic and commentary from various online sources including webpages (corporate, personal and technology focused), social networking sites such as Quora, Facebook and Twitter, online magazines, blogs, and online news media sites (hereafter referred to as articles). These were generated from a dynamically bounded review, allowing for the search to respond to the information returned. Google searches in English commenced in July 2017 continuing intermittently to late July commencing with the terms ‘poverty + programming’, ‘disadvantage + programming’, ‘poverty + software developer/development’ and ‘non-formal education + coding/programming’. Searches were conducted until the 10th Google page was returned, where relevant results generally diminished. Approximately 65 relevant ‘articles’ were returned from the initial search with occasional post-July searches identifying additional material, and some in Bahasa Indonesia. Articles were bookmarked and converted into PDF. These were archived using reference management software.

While the limitations of the ‘non-scientific’ literature explored in this paper arguably include a general failure to report long-term results, (mostly) under-theorisation, insufficient attention to the students’ voice, and with few exceptions little recognition of program limitations or instances of failure, they nonetheless arguably offer our project—in the absence of more formal academic publications in this area—a rich research resource providing access to internationally dispersed instances of current practice. We state ‘mostly’ under-theorised as there is some discussion around programming and poverty, referencing social theory, which surprisingly draws on Bourdieu’s Social Reproduction Theory; Byrne’s discussion of apps for low-income Americans exemplifies this (2014). We also draw upon Bourdieu to not only interrogate and understand these instances of practice, but to also—as a result of our analysis of such—inform initial thinking around the construction of our own coding curriculum. In conceptualising this project, we felt that we needed a social theory framework that would allow us to better understand the students’ current socio-economic position, how this might inform their relationship to the coding curriculum, and the kinds of ‘agentic’ learning experiences the curriculum might offer.

With the seemingly positive contextual case identified above, and indeed affirmation by Indonesian state policy^5^, discussion below reviews online literature reporting instances of existing practice in providing coding classes to the disadvantaged - as read through the lens of Bourdieu’s social reproduction theory.

## Results

One of the students commented that the reason they are not motivated to study programming is because they have not seen many programmers in Tanzania. They then fail to make a connection to the future of the course they are studying. This appears like a dark future, or venturing into the “unknown” destination. The future appears to be dark (Oroma et al. 2012. p. 3822).

### Bourdieu and existing practice

Bourdieu’s social reproduction theory is concerned to account for why individuals (as members of groups) are not only located within particular socio-economic positions, but act in ways seemly reproducing their position. Central to social reproduction, however, is the habitus, which Bourdieu refers to as ‘the schemes of perception, thought, and action’ (1989, p. 14). As a ‘blueprint’, it comprises one’s sense of place, and mental structure through which individuals apprehend the social world. It is a result of the internalisation of the structures of their world, and the kinds of capital associated with those (pp. 17–18). The habitus is tied to the ‘perception and appreciation of practices, cognitive and evaluative structures which are acquired through the lasting experience of a social position’ (p. 19). As Yang Yang explains, the habitus is our thoughts, our perceptions and dispositions (2014, p. 1525). The habitus is relational. It is experienced in relation to others who broadly share the same habitus (Bourdieu 1989, p. 19) for whom it comprises ‘common sense’ (p. 19). Hence, an upper class environment will likely engender a different habitus compared with a lower class environment; resulting in the possession of different dispositions, common sense and aspirations (Bourdieu 1996, 1989). Indeed, an online answer to the question: Why do not more poor people learn programming, explains this differential aspect of the habitus particularly well. The answer being: ‘the same reason a lot of rich/middle class people don’t learn to code, because they don’t want to and/or have better things to do’ (Mahajan 2014). Though Bourdieu’s notion of habitus can be critiqued on a number of grounds as identified later in the discussion of limitations, it arguably provides a view of an individual’s disposition and behaviour going beyond a narrow focus on the economic to include the socio-emotional impacts of such. And with this, the notion of habitus aligns with more critical understandings of poverty and disadvantage.

While poverty can be understood from a largely quantitative perspective measured in terms of income level (Soergal 2015) (fixed or relative to societal expectations), a capacity to obtain things or fulfil basic needs (Hubpages 2014; Handayani 2012), or consume a number of calories (Headey and Ecker 2013), these approaches fail to capture the *experience* of poverty (subsequent disadvantage) and the socio-emotional impact. Humans have psychological needs that, not unrelated to food and other economic resources, are located within a web of socio-cultural relations, expectations and obligations (Lister 2004). Quantitative approaches to understanding poverty and disadvantage can overlook this and the extent to which a lack of participation, which is often the result of social exclusion informs the experience of poverty (Sen 2000). Poverty is thus also socio-cultural (Lister 2004, p. 22, Sen 2000). As Byrne (2014) notes in discussion of building an app for the disadvantaged, ‘living on a low income translates into other forms of scarcity: of power, information, respect, opportunity, time, health, security, and even of sleep’; thus, poverty also restricts a person’s ability to make choices about their life (Nussbaum 2011; Habibis and Walter 2009; Sen 1999). Bourdieu would reference this outcome in terms dispossession, which is the inability to directly speak back to power arising from being dispossessed of capital and authorised discourses (Bourdieu 1991). What is more, the disadvantaged know they are disadvantaged being equally aware of the less than favourable perceptions of others (Kelly 2015). Notwithstanding the significance of other forms of capital in the experience of poverty and disadvantage, our project does not dismiss the importance of economic resources or capital; those ‘immediately and directly convertible into money and may be institutionalised in the form of property rights’ (Bourdieu 1986, p. 16). Indeed, we hope that our students’ acquisition of programming skills will translate into increased access to economic resources.

Poverty in Bali is often directly related to a lack of economic capital resulting in levels of extreme poverty in some instances (The Bali Times 2012). For example, the percentage of children living on less than $2 per day in 2010^6^ in various (rural) regencies (as measured by the Indonesian basic needs approach) was particularly high: 71.52% for the Karangasem district and 64.3% for Bangli. More disconcerting is that over 7% of children in Karangasem were reported to be living on less than $1 per day (SMERU 2012, p. 267). Indeed such is the centrality of economic poverty to the experience of disadvantage that the majority of coding classes for the disadvantaged that are explored below, are established with the aim of enabling students to seek improved employment futures within the field of software development—a field in which they are currently under-represented (Coleman 2016). For example, in noting the disparity between the number of ‘computing jobs’ available in the USA and the number of graduates in computer science, refugeecodinghut.com, explains that:

One of the core pillars of our giving strategy is livelihoods, i.e., initiatives which empower individuals and communities to lift themselves out of poverty by creating sustainable income opportunities. Teaching computer science fits perfectly within that strategy, as it addresses one of the core needs of a community of over 50,000 refugees in the State of Utah (2015).

Nonetheless, the contexts framing disadvantaged students’ participation in free coding classes seem amenable to understanding through Bourdieu’s notion of habitus. The habitus, it will be recalled, comprises our thoughts, perceptions and dispositions (Yang Yang 2014, p. 1525). It is experienced in relation to others who broadly share the same habitus (common sense) as a result of participation in similar fields (Bourdieu 1989, p. 19). A lack of previous access to a field can mean that an individual is not well-disposed to effectively participate within it when the opportunity arises. The online literature also report not only disadvantaged economic conditions inscribed by a lack of access, but the social-emotional conditions and consequences in terms of limited and limiting student aspirations. Disadvantaged groups identified in the literature include Native Americans (Shannon n.d), girls (Black Girls Code 2017), Latinos, African Americans, at risk youth (Chmelewski 2015), the rural poor (Mader 2014), urban poor (CBS 2015; Patane 2015), Asian Americans (Coleman 2016), prison inmates (Jackson 2015), refugees in the USA, and those in international contexts including India (Doshi 2016), Madagascar (Mulligan 2017), Ghana (Herz 2013; Walling 2005) and Pakistan (Qasim 2016). And though not all contexts providing coding classes are inscribed by the same conditions, many are characterised by socio-economic brokenness and its effects (Chmelewski 2015). As Green explains with reference to poverty in Rwanda:

People who endure extreme financial scarcity experience brokenness in multiple ways. So we should not be surprised, really, to read how people living on less than $2 per day responded when asked, “What is poverty?” Poverty is …. An empty heart. Not knowing your abilities and strengths. Not being able to make progress. Isolation. No hope or belief in yourself. Knowing you can’t take care of your family. Broken relationships. Not knowing God. Not having basic things to eat. Not having money. A consequence of not sharing. A lack of good thoughts (2014).

A Memphis-based teacher providing coding classes to minorities and girls does so against a backdrop of decades of ‘children growing up in communities such as Binghampton … surrounded by blight, crime and poverty’ (Coleman 2016). Elsewhere a programmer teaching children from poor households reports ‘many of the kids I teach live in slums. People pee on the stairwells. Graffiti adorn the walls. Common areas are littered with uncollected rubbish, and it’s the people who are living there who are doing it’ (Chew 2014). Other programs not so inscribed by crime or violence, nonetheless, report a lack of home infrastructure and access to economic resources needed to effectively engage in study of software development, including books (Walling 2005), affordable stable internet connections (Walter 2015; Mader 2014), Wi-Fi (Doshi), a computer (Bhowmick 2016), or tuition (Elliot 2016). In some contexts, a lack of access is compounded by gender—males receiving preferential resourcing (Doshi 2016). Moreover, a lack of economic capital is often associated with a lack of time to invest in formal study, arising from family obligations and multiple low-paying employment commitments (Doshi 2016; Byrne 2014). Doshi adds that parents in disadvantaged contexts may not necessarily support children taking up coding classes, especially girls tasked with housework (2016).

Significant in terms of the habitus, as a disposition, are the comments of Chew observing that with students:

Resigned to a life time of menial work or crime … it takes a lot of work to even get them to acknowledge that there are other options, and even greater effort to get them to work towards those options. These kids and many more like them will grow up inheriting these self-limiting mindsets, and will likely never grow out of it. A large majority will be the next generation of hardcore poor (2014).

Chew’s reference to inheritance is poignant not only because it highlights Bourdieu’s argument that the habitus is in part acquired through birth into existing relationships, but also because as Bourdieu reminds, its apparent ‘naturalness’ is none other than a consequence of ‘history turned into nature’ (Bourdieu 1977, p. 78). Bourdieu maintains that children inherit capitals held by their parents and associated habitus, such that while a child may inherit economic capital, they may equally acquire the family’s cultural capital (1996), which can be converted into economic capital. The challenge often faced by the disadvantaged in formal education is that which arises from the assumption that success is the outcome of merit and not the student’s inherited capital with which the curriculum is often implicitly aligned.

The online literature reports that the experience of poverty and disadvantage in contexts inhabited by students in coding classes for the disadvantaged is variously associated with a soul crushing hopelessness, helplessness and apathy (Chew 2014). Students initially had little to no long-term goals (Chew 2014) reporting feeling like trash (Kelly 2015). Previous failure was a common experience leading to a lack of inspiration and confidence to even believe success was possible (Walter 2015; Chew 2014). Students were easily discouraged resulting in easy abandonment of any career possibility (Walter 2015). In some contexts, begging was the ‘logical option’ providing an income solving the more immediate problems of hunger and malnourishment (Gamesbrainiac 2014).

But whereas economic capital might be best understood as ‘money ‘in its simplest sense, social capital is perhaps best understood as a social network or group in which an individual is part. It is:

The aggregate of the actual or potential resources which are linked to possession of a durable network of more or less institutionalised relationships of mutual acquaintance and recognition - or in other words, to membership in a group -which provides each of its members with the backing of the collectively-owned capital, a “credential” which entitles them to credit, in the various senses of the word (Bourdieu 1986, p. 21).

Colloquially known as the ‘old school tie’, it is a ‘currency’ recognised and exchanged among a social group, though ‘not a natural given, or even a social given’ (Bourdieu 1986, p. 22). Rather, it is the consequence of production, mutual recognition (of the exchanged capital) and reproduction; the product of:

Investment strategies, individual or collective, consciously or unconsciously aimed at establishing or reproducing social relationships that are directly usable in the short or long term, i.e., at transforming contingent relations, such as those of neighbourhood, the work-place, or even kinship, into relationships that are at once necessary and elective, implying durable obligations subjectively felt (feelings of gratitude, respect, friend-ship, etc.) or institutionally guaranteed (rights) (1986, p. 22).

Possession of the right kind of social capital ‘profits’ the holder in ways which can be material or symbolic (Siisiäinen 2000). Of significance in terms of poverty is that its absence negatively impacts the individual’s capacity to engage ‘social customs, activities and relationships’ (Lister 2004, p. 22), such that poverty and disadvantage not only constrain investment in maintaining social capital, they limit opportunities to expand and acquire social capital. As noted above, Indian developers benefited from social networking with western counterparts (Heeks 1999).

Of interest here are Byrne’s (2014) observations regarding social capital. Not only does Byrne specifically mention ‘social capital’, she explains that it is as significant to success as economic capital:

It also became clear that inequality isn’t purely about income. It’s about information and status and opportunity. If you look at the dollar amount, my own income as a freelance writer probably wasn’t much higher than some of my interviewees, but I still had resources like educational credentials and social capital which many of them lacked. One of the reasons that graduation rates among low-income first generation college students hover at around 10% is that they don’t have the “college knowledge” taken for granted by their peers.

Though with exceptions, the online publically available literature shows that coding programs for the disadvantaged are not well theorised, they nonetheless provide what might be considered evidence of the significance of social capital, mostly through referencing the absence of role models in the lives of disadvantaged students. Students are reported to have never known a programmer (Ramsey 2016), or have never seen a programmer of their colour, ethnicity or background (Walter 2015). Nicks reports, for example, that ‘in 11 (US) states last year, not a single black student took the Computer Science Advanced Placement Exam for college credit’, suggesting that software development has become privileged knowledge for the groups that do enrol (2014). Denver’s reference to males acknowledges what is seen as the exclusionary ‘bro culture’ of computer science (Collins 2017), in response to which there have emerged a number of free coding programs for girls and women specifically (Bradord 2015). Elsewhere, students report not having anyone with which to even discuss the possibilities in coding (Bhowmick 2016). This lack of social capital translates to the field of software development being experienced as alien, foreign and intimidating, eliciting an almost self-exclusionary response by the disadvantaged (CBS 2015; Baker and White 2013).

In the same way, the provision of coding classes for the disadvantaged seeks to ameliorate a lack of economic capital through provision of economic resources; the online literature reports that they often seek to offset a lack of social capital, providing students with links to the software development field. Typically, this involves visiting software development sites (Baker and White 2013), work experience opportunities (Jackson 2015), making connections with employers (Walter 2015), using teachers from the students’ background (CBS 2015), and mentorships (Hulburt 2016; Heim 2012). Acknowledging the value of access to social capital, Williams, a coding teacher of low income students in San Francisco who grew up in ‘the projects’, notes:

There is something important that happens when kids of color can see someone like themselves in leadership positions. Something powerful; which is why it is important that someone like Stevon Cook is CEO and Joe (CBS 2015).

Stevon Cook, CEO, is also a man of ‘colour’ from ‘the projects’ and like Williams he maintains it is ‘vital that these kids see talented people of color working in the tech industry’ (CBS 2015).

Such is the value of social capital that the online literature also reports concern among some in the software development field with the possibility that it is to be inhabited by those traditionally excluded. In a post responding to commentary around President Obama’s call for all to learn to code, Loukides (2014) highlights his amusement ‘by the anxiety among certain programmers about the great unwashed getting into their privileged domain’. He adds: ‘an overpopulation of coders? That isn’t going to be a problem’. Like Obama, his view is that all should learn to code, not because all will code as a career, but to open opportunities to ‘those people who haven’t been programming since they were 10 years old’; to disadvantaged groups in whose career trajectory Wal-Mart, McDonalds and the minimum wage figure prominently. Here Loukides supports Anil Dash’s (2013) response to Obama detailing that the plan requires not only learning code but also the capital that ‘works’ in the field of software development, as the ‘people in power in the tech industry right now, [are] not inclined to let others in’. He advises that if you are one of those others, you must understand the language they are speaking (2013). This cautionary advice points to issues of access to the field and its social capital. It also points to the possession (or lack thereof) of cultural capital, and the *doxa* associated with the field.

Cultural capital is that ‘which is convertible, in certain conditions, into economic capital and may be institutionalised in the form of educational qualifications’ (Bourdieu 1986, p. 16). In the embodied form, it comprises ‘long-lasting dispositions of the mind and body’ (Bourdieu 1986, p. 17). Embodiment references the assimilation of capital over time through investing in particular kinds of culture (p. 18). Although it can also be ‘inherited’ (Bourdieu 1996, p. 12), cultural capital can be attained through education credentials associable with entrance into typically middle to upper middle class fields (Bourdieu and Passeron 1990, p. 227).

For Bourdieu, the field is a location, domain or ‘arena’ wherein individuals are positioned in a relationship with others according to the rules (of the game) informing its structure; capitals exist only in relation to a field (Bourideu and Wacquant 1992, p. 101). As noted earlier, these rules, internalised as the habitus, can also be understood as *doxa* or the socially shared meaning that informs how a context is read. Within a field ‘*doxa*’ (as a sum of capitals) comprise the:

Unquestioned shared beliefs which constitute fields and is an act of symbolic power in which the accumulation and distribution of capitals explains which beliefs and truths, which practices, distributions, hierarchies or sets of social relations are considered “natural” or appropriate (Hastings and Mattews 2015, p. 549).

In the field of software development, entry has often been and often still is on the basis of a university-level qualification (cultural capital), a computer science degree or equivalent (Indeed.com, 2017b). With the proliferation of online information, this entry mode is challenged through more opportunities to be self-taught (Jackson 2015), and the growth in organisations providing fast-track pathways into the industry, such as the ubiquitous coding bootcamp (Switchup 2017). Additionally, there are also the kinds of opportunities which this project aims to establish. Often the work of individuals, small groups, and sometimes corporate social responsibility are invariably staffed by volunteers, exist outside of formal education, and are sometimes located in less than salubrious accommodation, like tin sheds (Skees 2017) and laundromats (Kavilanz 2016).

Black Girls Code is an example of this model referred to by Loukides, who noting that ‘there’s no “Black Girls Practice Law” or “Black Boys Bank.” Law, banking, medicine—any professional discipline except for software’ (sic), draws attention to the extent to which credentials (cultural capital) can still exclude minorities from certain fields. Even when the significance of access (by a Degree) is diminished, the disadvantaged may find participation in the field remains constrained by relations of power associated with *doxa*. As Bourdieu notes, relationships within a field are defined by power and struggles around the accumulation and distribution of capital therein (Bourdieu 1991, p. 242; 1986, p. 20), such that where capital is lacking, as a result of unfamiliarity with a field, the individual with limited power over the rules of the game may experience failure (Erel 2010).

## Discussion

### Cautious optimism

“It’s changing their lives. It’s an amazing story to tell, and Memphis is right in the center of it (Coleman 2016)”.

With Loukides and Dash highlighting the constraints upon access and (successful) participation faced by the disadvantaged, it might be asked: Is it even possible for the disadvantaged to succeed in software development? Our view is that though difficult, it is possible. The online literature identfied above show that most coding programs share the goal of enabling students to seek improved employment opportunities and lifestyles. Referencing a program located in a US public housing development, Chmelewski (2015) for example, reports its goal is to ‘break[ing] down the socio-economic barriers … keeping so many kids away from technology and future life opportunities’. Similarly CodeON in South Carolina aims to give children ‘access to basic tech skills that will better prepare them for their future in college or in a job’ (Kavilanz 2016), while C4Q: Recruits New Yorkers from low- income, underserved communities, teaches them programming over an intensive 10-month course, and then helps them land jobs at companies like Pinterest and Kickstarter (Peters 2017). With these aims, coding programs for the disadvantaged not only aim to facilitate change, they are implicitly working on the premise that individuals have a capacity for agency, such that we maintain, here too Bourdieu holds out possibilities for understanding existing non-formal education based coding classes for the disadvantaged, and for framing our program in Bali, though as Yang Yang (2014) notes, his work around agency is not as convincing. Our view, however, is that there is in Bourdieu’s work, space for the conceptualisation of agency, and thus there remains the possibility that education can support the exercise of agency and success. This (possibility of agencey) is then, our starting point for the discussion of the results of the online review of practice identified above.

Like Yang Yang, our response to Bourdieu’s claim that the working class can never achieve success in education is that: ‘our real-life experiences tell us this could not be more wrong’. So too does the story of Stevon and Joe above, and (paradoxically) Bourdieu’s own educational success (Yang Yang 2014, p. 1528). Central to identifying possibilities for agency in Bourdieu’s work and for our proposed coding programm is the notion of habitus. While intended to overcome typical sociological dichotomies/binaries including that of agency versus determinism, the difficulty for Bourdieu is that if the habitus (as a ‘system’ for how individuals perceive the world) resides below the consciousness (1989, pp. 14–19), how is it possible for an individual to be other than a determined product of their habitus? And while it appears Bourdieu’s initial conceptualisation of ‘reflexivity’ provides a space for agency, Bourdieu initially viewed reflexivity as a capacity mostly belonging to himself and other 'appropriately trained' sociologists (Yang Yang 2014; Mesny 2002). Akin to the Marxist ‘expert’s’ capacity to see false consciousness where workers could not, this seems a self-indulgent reflexivity, exemplifying one of Baudrillard’s key arguments against the value of critical theorists: “This is the trap of critical thinking that can only be exercised if it presupposes the naivete and stupidity of the masses” (1995, p.81).

Yang Yang’s review of Bourdieu and agency also explores possibilities for change inherent in his notion of ‘hysteresis (effect) of habitus’, which Bourdieu defines as ‘one of the foundations of the structural lag between opportunities and the dispositions to grasp them’ (1977, p. 83). But here Bourdieu is unclear around the timing and duration of the needed ‘crisis’ that causes the habitus to ‘generate non-adaptive forms of behaviour’ (Yang Yang 2014. p. 1530). Second, there is in Bourdieu a lack of clarity around what ‘causes people to become resigned and what results in revolt’ (p. 1531). As noted, poverty and disadvantage often result in resigning to circumstances and not pursuing longer-term opportunities (Marinos 2016). It also remains unclear regarding the extent to which rational/reasoned/objective choice acts in circumstances of crisis, and here Yang Yang (2014, p. 1531) rightly questions Bourdieu’s seeming assertion that only those agents trained in academia may in fact do so. But what is rational? Is begging a rational choice? Yang Yang thus holds that while hysteresis ‘explains the uncertainty, confusion and frustration that arise when social agents experience a change in a given field …’ it remains unclear how rational choice can overtake habitus and guard or seek individuals’ positions in such a changing environment (p. 1531), such that the notion of hysteresis insufficiently explains change and agency.

Though in contrast, Courtney’s findings from a study of school leaders’ responses to neo-liberal corporatisation of UK education (Courtney 2017), identify agency in the dynamic of hysteresis, it should be noted that this was not by adapting to the newly reconstructed field of healthcare, but in resistance through maintaining the existing habitus. Courtney proposes that Bourdieu’s notion of hysteresis be extended to account for three outcomes of field restructuring. The ‘agent’ may (choose) either (1) try to force field conditions to return to those aligned with their habitus; (2) attempt to re-align the habitus with the field, or (3) ‘remain discursively and materially de-privileged through their reduced capacity to play the game according to the field’s new rules’ (p. 1064). While in our view the third option seems the antithesis of agency, we continue to ask where the presumably ‘rational choice’ comes from if all available choices are structured by field and habitus? Is there something beyond to which individuals have access? If for example, those endowed with the kind of habitus towards which the reconfigured field is headed, then the decision to self-align seems more a matter of working with an existing blueprint (habitus). Thus it seems that either (1) the individual is suffering from the illusion of agency (choice) (Yang Yang) or (2) the notion of habitus is insufficient—being overly rigid. Our view is the latter. We acknowledge the undoubtedly strong influence of the habitus, but suggest that the information/knowledge boundaries upon which it is constructed are perhaps more porous than a less-tolerant reading of Bourdieu envisions. It may be beneficial to approach such structuring knowledge in terms of dynamic continuums of relative priority. Indeed in Bourdieu and Wacquant’s (1992) assertion that the habitus is ‘an open system of dispositions that is constantly subjected to experiences’ being constantly reinforced or modified by them, we find a space for this reading (p. 133). Moreover, as Bourdieu and Wacquant assert, the habitus ‘is durable, but not eternal!’ (p. 133).

In response to these limitations, Yang Yang (2014) looks for agency in Bourdieu’s notion of ‘deviant trajectory’. This particular notion arguably holds promise for facilitating agency in a coding class for the disadvantaged; with students who may have never anticipated interaction with the field of software development. Bourdieu holds that a deviant trajectory is one wherein there is a ‘misfire’ in ‘the homology between positions and the dispositions of their occupants’ (1996, p. 183). Though conceptualised in the context of elite school students taking up the ‘pole opposite to the position to which they were (by virtual of their existing habitus) promised and which was promised to them’ (p. 184), the concept holds that the greater the (relational) distance between the two (fields)—the new and the old—the greater possibilities for agency (p. 184). Though as Bourdieu also notes, this can result in instability within the new field, ‘failure’ and a reversal of direction (pp. 184–185). But in those occasions of success, Bourdieu seems, nonetheless, to suggest that this too is a product of the original habitus. Though again this is a conclusion associated with the privileged who hold to a habitus in which perseverance and ‘no right to failure’ are valued expressions of capital, ‘They are condemned [by their habitus] to excess, to extremes, to bold ostentation, which alone can justify their renunciation of temporal certainties’ (186). Still, Yang Yang sees possibilities in Bourdieu for education to support agency, as we also do. While Bourdieu was critical of education’s capacity to simply reproduce existing advantage and disadvantage (1996), he equally saw opportunities to intervene if education would be universalised. His later work extending reflexivity to the ‘people’, viewed universal public education as significant to this (Mesny 2002, p. 65).

In contrast to the narrower interpretations of Bourdieu and agency, the publically available online literature shows that non-formal learn-to-code opportunities can support student agency and produce successful outcomes. The article, ‘Meet the Non-Typical Programmer Who Beat Poverty and Shattered Stereotypes, for exampple, reports how a girl familiar with poverty, now has a work portfolio on LinkedIn and has ‘developed a strong professional network of friends and colleagues all over the world’ (King) - all indicative of acquiring “capital”. Skees (2017) reports on a Bangladeshi girl, Nila, born into poverty that ‘might have followed in footsteps of her parents for a lifetime of arduous manual labour and chronic hunger’, but with the help of free education including coding, has the confidence to dream of well-paying creative work: ‘By the time I finish schooling … my mother will be old. So I will design a robot to do her housework for her, and I will take care of her’. It is also reported that Lakota Sioux Indian students in free coding classes on the Pine River Reservation, who ‘never knew they were good at anything, not only “love” the learning, but “the impact [on] younger kids has been really obvious” (Shannon n.d). This article reports that one of the students is ‘now a sophomore in high school, and one of the best students’. Moreover: She won the best animation prize … despite never having heard of computer animation. It inspired her to become an animator and she’s now looking for college programs in animation (Shannon n.d).

Elsewhere, Emmanuel Escamilla, founder of the CodeX program ‘designed to teach codding as a way out of poverty’, explains that ‘70 precent of the kids in our first cohort didn’t have internet access at home. Six months later, they won the congressional app challenge’ (Burt 2016). While we could highlight more instances of success such as EdTech in Pakistan (Qasim 2016), the success of Stevon and Joe from MissionBit suggests first, Bourdieu is right in terms of the impact the habitus can have upon children’s life chances, and second, that change and agency are possible. Stevon notes that not only was his ‘world completely changed when … exposed to some great opportunities,’ but this exposure would generate his mission some 12 years later, ‘to go into communities where they would never even consider a computer science course and spark something in the minds of low-income students’ (CBS 2015).

However, with the possibility that agency can be generated through the unfamiliarity of a new field, as the exemplars of online practice identified above seem to be also showing, Yang Yang's recommendation of a non-traditional approach grounded in explicit pedagogy seems to provide a 'workable' option for curriculum construction in the proposed coding program. That is, a pedagogic approach, which is planned, strategic, scholastic, and experimental, or rather, practice-based in which an individual is ‘fully aware of the available resources and can be reflexive all the way through until a secondary habitus is constructed’ (2014, p. 1533). But to this we would add that reflexivity must be grounded in engaging how relations of power inform the context, student and curriculum relationship (Tristan 2013), not necessarily in an adversarial sense, but through appropriation – and the acquisition of capitals. This when considered in relation to the reading of practice above, suggests that our curriculum will need to not only provide access to code and capital resources such as hardware, software and the Internet, but also to opportunities to identify access and engage the social and cultural capital of software development. We are for example, already talking to business in Bali to establish linkage opportunities. Additionally, the proposed curriculum should ideally also be taught (or co-taught) by teachers who are ‘local’ who have a similar ethnic if not class background, who might provide valuable role models. The curriculum might align with existing Indonesian or international qualifications pathways and importantly provide access to the *doxa* of the software development industry in Indonesia and beyond. We maintain that not only are all of these strategies possible, they would largely replicate strategy underpinning existing curriculum areas at the Slukat Learning Centre. But the current SLC curriculum not only delivers subject specific disciplinary knowledge, it also provides opportunities for students to access the kinds of social and cultural capital that they otherwise have little to no access to. Our coding curriculum aims to fit into this current approach, which has proven successful in terms of student vocational outcomes. This existing SLC curriculum is also supported by a strong focus on local wisdom, framed by the Balinese notion of Tri Hita Karana,^7^ comprising:

1. Harmony Among people (*Pawongan*). This is implemented through developing student character through a leadership program, English and basic computer classes, and working to improve student confidence through enabling interaction with international volunteers.
2. Harmony with Nature/Environment (*Palemahan*): This is implemented through creating awareness and understanding about the importance of the environment. Students are involved in a recycling and organic foods program, beach cleaning and a no plastic bags program.
3. Harmony with God (*Parhyangan*). Students engage in local wisdom programs, yoga, and *Tirta Yatra* activities such as excursions to temples, and learning how to make offerings (*sajen*).

Our coding curriculum will also need to be framed by the principles of *Tri Hita Karana*. With this, we see opportunities to work with the students and the community to develop applications and software that are culturally relevant, responsive and culture sustaining. And while this has not been a discussion about specific software development technologies, curriculum content or pedagogy, we acknowledge that the online literature, like the formal literature, emphasises learning through fun activities such as game development (Kayser 2013; Patane 2015) and project and group work (Walter 2015, Feldman et al. 2003). It equally advocates providing as much learning material as possible (apps, programmes, videos, books, slides) on redistributable USBs for use when the internet is not at hand, allowing for offline coding (Giantsparklerobot 2016). At this stage, however, we envision being able to offer students access to C# and the .Net Framework, web development technologies (such as CSS, HTML and JavaScript), PHP, Android development (using Xamarin) and possibly some Unity work.

#### Limitations

But despite our sense of optimism emerging from the contextual case and the success stories revealed in the literature, our project is not unaware of key challenges and 'fundamental' limitations that have a potential to impact its successful implementation. These include not only the critique of Bourdieu, but also those facing the Indonesian software development sector as a whole. With regard to the former, we acknowledge that Bourdieu is criticised for being imprecise regarding what comprises the capitals; a consequence of which has been inconsistent research findings in projects using Social Reproduction Theory (Sullivan 2002 146). Our project, does not however, aim to generate a list of students’ capitals wherein ‘evidence’ might be identified to demonstrate the veracity of Bourdieu’s theorising. Rather, we reference Bourdieu to guide building a curriculum that avoids the uncritical liberal-individualist assumption that because all are 'theoretically' or in principle equal, that all are equally positioned (in practice) to achieve success. The results of the online literature reviewed above clearly show this is not the case (Collins 2017; Mader 2014). Instead we draw upon Bourdieu to understand where students may have come from, what their understanding of the field of software development might be, and what might be needed to help them achieve success in this field. Importantly, we also draw upon Bourdieu to understand the possibilities for agency that might be engaged by the disadvantaged in entering a field with which they may have had little to no previous contact. Moreover, while some see in Bourdieu a lack of clarity around the treatment of agency as we also do, like others (Yang Yang 2014; Nash 1990) we also find support in his work that validates offering students’ access to what may be an alien and possibly intimidating field. Hence, while we hold to the ideals of liberal-humanism, we acknowledge their limits - in practice - such that our view is fundamentally and critically sociological.

We note a further critique of Bourdieu concerns his inability to account for individuals who achieve success despite a lack of cultural capital (Nash 1990). Here we not only acknowledge the aim of sociology is to understand the social as opposed to the individual, we also note that Bourdieu did not preclude attempting to understand why, for example, individuals within classes who with interrupted trajectories, acted in ways contrary to ‘natural’ class expectations (1996, pp. 183–187).

Notwithstanding the limitations of Bourdieu's theoretical tools, there are those associated with the Indonesian software development sector and ICT Education, which are neither insignificant nor unacknowledged by the Indonesian government. BEKRAF adds:

On the other hand, this sub-sector still faces various challenges, among them human capital/resources both in number and quality, minimal industry investment, and insufficient protection recognising the importance of domestic developers. This situation makes this sub-sectors’ environment insufficiently developed (2017).

While many of the challenges in Indonesia are those faced by other developing countries growing a software development sector (BMZ 2011; Bamiro 2007), among which can be relatively low wages in some instances (Fendy 2016; Andre 2015),^8^ the Indonesian government is not unaware of such, having committed to better supporting software development.

… BEKRAF can undertake a number of actions. It can, initiate the emergence of incubators for applications and game development. Place the right elements for application and game development in education and protect local developers and support them in promoting their field (BEKRAF 2017).

While BEKRAF policy is clearly positive, overall Indonesian policy and practice around education as an institution to support not only ICT creativity but also software development, is mixed. While policy voices laud ICT and software development (Intan 2016), ICT has much to the chagrin of those in the industry been removed as a stand-alone curriculum area in schools (Susanti 2016; Vota 2014). Moreover, we acknowledge President Widodo’s own caution around the potentially negative impact of digital technologies upon youth:

The effect of information and communication using our smartphones is that they can erode the value of character among our children (sic) (Marwati 2017).

We also acknowledge that both household and national infrastructure is often inadequate to support the kinds of learning experiences that could be associated with a stand-alone curriculum. And while policy seeks improvement in school and higher education outcomes (Chang et al. 2014; Smith 2017), questions remain about the quality of graduates, including those from Indonesian programming courses (The Australian 2012; Global Business Guide Indonesia 2016). Indicative of this concern, Abud explains that in Indonesia ‘most [software development] companies have to invest six months or so in training the talent they need, making scaling up a challenge’ (Abud 2012, p. 11).

Still, there are other limitations and constraints. We are aware of the argument which holds that poverty does not respond well to programming. Some argue that the best thing a developer could do is to earn a high income and donate to the poor (Cthulhu 2011). Others highlight that even Google’s forays into poverty alleviation did not end well (Strom and Helft 2011). Moreover, we are aware of the argument that not all can program (Baduser 2014; Lee 2014). Together, these dynamics remain formidable challenges. Indeed, changing the lives of the poor is itself a long term process and some might argue that a globalisaing neo-liberal economic structure makes this all the more difficult (Tamatea 2010).

## Conclusion

Our project, however, is not aiming to challenge the global economic order, as much as to equip disadvantaged rural Balinese youth with coding skills that may better support them to participate in this order. Ours is a micro-level focused project, aware of broader macro-level socio-economic structuring, but which has as its domain of potential change, the local and in particular the individual – as a member of a socio-economic group. Our aim is to offer coding lessons as part of a larger non-formal education curriculum at the Slukat Learning Center in Bali with the hope of facilitating enhanced agency among the disadvantaged. With this, we acknowledge the capacity of education to facilitate emancipation, be it formal or non-formal, and we acknowledge the role of education in supporting the supply of developers to a developing software industry (Arora and Gambardella 2008). We further acknowledge the international movement of state governments to provide all students with access to coding in formal education (Bocconi et al. 2016), and like Ruseva and Rissola (2016) hold that disadvantaged students should equally have access to this resource. We are not unaware, however, of a view that capital (industry backed by the state), seeking to reduce developer wages, is behind the coding for all in schools movement (Bresnihan et al. 2015).

Still, we argue that coding should be available to all not because all can or will code. Rather, because in an era inscribed by intensifying abstraction (Baudrillard 1995), all should be given the opportunity to code. We understand that established software producing nations dominate globally, and this will likely hold for some time, such that developing nations will continue to consume off the shelf products, though perhaps increasingly in the form as Software as a Service (SaaS) (Bartels and Giron 2017). Yet in this space local developers are emerging to produce products meeting local cultural and language requirements (Bartels and Giron 2017). The global corporate sector is not unaware of the localisation need (Bartels and Giron 2017) and in Indonesia the *GoJek* start-up is an example of this. The local (national) creative economy as signalled by BEKRAF equally provides opportunities for localised software development (Boccella and Salerno 2016).

Our project is based on the premise that the need for software developers in Indonesia should only grow. In formulating this premise as a ‘justification’ for the coding curriculum, we have been guided by the sociological work of Baudrillard and the literature pertaining to the context of growth in software development at global, national and local levels. There is, we maintain, arising from both the theory and the attendant data a case for the value of such a curriculum. With software developer shortages reported internationally (van Heur 2016; Marnane 2016; Min 2016; Tao 2016; Fiscutean 2015), our project sees opportunities for individuals in free-lancing, opportunities to work for local companies, opportunities to work for international corporations, and even opportunities to work in international settings. SLC students equipped with ‘character’ derived from local wisdom, a command of English, capital from interaction with both Eastern and Western tourists, and coding skills, may be well placed to meet this shortage or perhaps even win scholarships for enrolment in tertiary level computer science courses, nationally and internationally. While success can undoubtedly be measured otherwise, in a context where many live on a few dollars a day, we see securing sustainable economic capital as a strategic priority and indicator of success.

Achieving this will, however, not be easy, not the least because of the challenges detailed above. But more than this, we know that possession of a degree in Bali is certainly no guarantee of middle class employment. Experience tells us that many young people hold degrees, but remain in labouring positions or those servicing the tourist industry at lower levels. Moreover, in Indonesia, both access to certain kinds of employment and indeed promotion can require the employee to first pay the employer. Furthermore, while we acknowledge that although Bali has become a developer ‘hot spot’, these claims need to be interrogated in terms of the national and ethnic composition of the developer cohort. While globally mobile digital workers may be able to afford the time and cost of moving from their metropolitan centres to ‘paradise’, to co-work and network with those similarly well-placed, we are not convinced that the Balinese themselves are equally represented in such spaces, beyond predominantly servicing such. But again, our project’s aim is not to challenge or subvert such flows. Instead it is to provide disadvantaged Balinese youth with more agency so as to be able to access these flows.

Finally, our project aims to provide opportunity to those who otherwise may not have had access to the field of software development, and this paper comprises a first step towards that goal. Our review of the publically available literature around current practice in the provision of non-formal education coding classes to the disadvantaged, not only reveals what is considered to be the obstacles to success, but also what is needed to achieve success in the software development field – at least at entry level. While most programs identified in the online literature view success in terms of equipping students with employment related skills, as we do, the literature also shows that success requires more than acquisition of code skills. With this, we have found Bourdieu’s Social Reproduction Theory particularly amenable for reading and understanding current practice and thus for framing initial thinking about our own coding curriculum. Bourdieu’s framework proposes that ‘success’ or being favourably positioned socio-economically is a consequence of the possession of a range of capitals, and our reading of the literature shows this to hold both in terms of why the disadvantaged are underrepresented in software development, and in terms of what is needed to redress this through the provision of non-formal education coding opportunities.

## Footnotes

1 Compound Annual Growth Rate.

2 Replacing the former Minister of Tourism and Creative Economy.

3 Translated from *Bahasa Indonesia*.

4 Bali’s capital city.

5 Discussion below acknowledges President Widodo’s concern about the negative impacts of digital technologies (Marwati, 2017).

6 Most recent data.

7 Three causes of well-being or three reasons for prosperity that include the balance between God, humanity and nature (*Parhyangan, Pawongan, Palemahan*).

8 To the contrary, the 2016 Kelly Indonesia Salary Guide reports that a software engineer with 5–8 years’ experience will earn between 5 and 8 million Rupiah a month (AUD $500–800).
